# Effect of Ethanol on the Metabolic Characteristics of HIV-1 Integrase Inhibitor Elvitegravir and Elvitegravir/Cobicistat with CYP3A: An Analysis Using a Newly Developed LC-MS/MS Method

**DOI:** 10.1371/journal.pone.0149225

**Published:** 2016-02-12

**Authors:** Narasimha M. Midde, Mohammad A. Rahman, Chetan Rathi, Junhao Li, Bernd Meibohm, Weihua Li, Santosh Kumar

**Affiliations:** 1 Pharmaceutical Sciences, College of Pharmacy, University of Tennessee Health Science Center, Memphis, Tennessee, United States of America; 2 Shanghai Key Laboratory of New Drug Design, School of Pharmacy, East China University of Science and Technology, Shanghai, China; Meharry Medical College, UNITED STATES

## Abstract

Elvitegravir (EVG), an integrase inhibitor for the treatment HIV infection, is increasingly becoming the part of first-line antiretroviral therapy (ART) regimen. EVG is mainly metabolized through cytochrome P450 (CYP) 3A4. Previously, we have shown that ethanol alters ART-CYP3A4 interactions with protease inhibitors thereby altering their metabolisms. However, as EVG is a fairly new class of drug, its kinetic characteristics and the effect of ethanol on EVG-CYPP3A4 interaction is poorly understood. In this study, we characterized EVG and cobicistat (COBI)-boosted EVG metabolism in human microsomes followed by ethanol-EVG, ethanol-COBI-EVG interaction with CYP3A. First, we developed and validated a simple, sensitive, and robust liquid chromatography–tandem mass spectrometry (LC-MS/MS) method for the quantification of EVG in the human liver microsomes. The lower limit of quantification for the drug was at 0.003 μM (1.34ng/ml). Extraction yield, matrix effects, drug stability, and calibration curves for the proposed method were validated according to the FDA guidelines. Time dependent kinetics data showed that 20mM ethanol decreases the apparent half-life of EVG degradation by ~50% compared to EVG alone. Our substrate kinetic results revealed that ethanol mildly decreases the catalytic efficiency for EVG metabolism. Inhibition studies demonstrated that EVG inhibits CYP3A4, and 20 mM ethanol causes a decrease in the IC_50_ of EVG. However, in the presence of COBI we were unable to determine these parameters effectively because COBI, being a strong inhibitor of CYP3A4, blocked the EVG/ethanol-CYP3A4 interactions. Docking studies predicted a shift of EVG or COBI binding to the active site of CYP3A4 in the presence of ethanol. Taken together, these results suggest that ethanol interacts with microsomal CYP3A and alters EVG-CYP3A4 interaction thereby altering EVG metabolism and inhibition of CYP3A4 by EVG. This finding has clinical significance because alcohol use is highly prevalent in HIV population, and there are no separate guidelines for these patients while they are on ART medication.

## Introduction

HIV-1 integrase strand-transfer inhibitors are the newest class of antiretroviral drugs that are used for the treatment of HIV infection. There are only three drugs—raltegravir, dolutegravir, and elvitegravir (EVG) that belong to this class of drugs [1]. As HIV-1 integrase exist only in HIV but not in humans the drug-mediated side effects are rare, which offer a favorable safety profile than the other antiretroviral drugs [2]. However, since EVG is metabolized primarily through cytochrome P450 (CYP) 3A pathway [3], there would exist a potential drug-drug interactions. In fact, co-administration with a strong CYP3A inhibitor such as Cobicistat (COBI) or ritonavir (RTV) has resulted in maintenance of high systemic exposure and prolonged elimination half-life. Although this is an example of favorable drug-drug interaction, there are potentials for unfavorable CYP3A4-mediated drug interactions comprising antiretroviral therapy (ART) drugs and other drugs that interact with CYP3A4. These interactions may lead to suboptimal effects of ART drugs and/or drug-mediated toxicity.

CYP3A4 is the major metabolic enzyme in the human liver and intestine that is responsible for the metabolism of nearly more than half of the available drugs in the market including ART drugs [4]. Inhibition or inactivation of CYP3A4 can cause mild to severe drug-drug interactions. ARTs such as non-nucleoside reverse transcriptase inhibitors (NNRTIs) and protease inhibitors have been shown to act as either inhibitors or inducers of CYP3A4 [5]. Inhibition of CYP3A4 by these ARTs may cause systemic drug toxicity, however some strong CYP3A4 inhibitors such as RTV can be used to improve the plasma exposure and half-life of other ARTs that are substrates for CYP enzymes. EVG administration causes induction of CYP3A and CYP2C9 in a dose dependent manner and inhibits CYP3A with high IC_50_ value (63 μM) [6]. Furthermore, co-administering EVG with some of the protease inhibitors and a newly approved chemokine receptor 5 (CCR5) antagonist such as maraviroc, substrates for the CYP3A, resulted in increased exposure of these agents to the system [6, 7] suggesting the therapeutic challenges associated with ART metabolism via CYP3A4.

Previously, our lab and other groups have demonstrated that alcohol consumption induces the expression of CYP3A4, as well as, ethanol alters the CYP3A4-ARTs interactions and metabolism of nelfinavir [8–11]. Specifically, ethanol binding to CYP3A4 active site via non-covalent interaction with the heme iron decreases the maximum spectral binding change for lopinavir and saquinavir [12]. Furthermore, ethanol exposure significantly decreased the IC_50_ values of amprenavir, darunavir, and nelfinavir but robustly elevated the IC_50_ of indinavir and ritonavir [12, 13], suggesting a differential impact of ethanol on the binding and bio-transformation of protease inhibitors. Similarly, our previous finding has shown that CYP3A4 is induced by ethanol treatment in monocyte-derived macrophages, important viral reservoirs for the HIV [8, 14].

As documented evidence reveals that alcohol consumption has significant influence on the effectiveness of ARTs, failure of treatment adherence, drug interactions, and hepatic and neurotoxicity [15–19], it is important to understand the effects of alcohol on the metabolism of EVG and its clinical consequences. Therefore, in current study we examined the effect of ethanol on EVG and EVG+COBI-CYP3A interactions as COBI is increasingly used as pharmaco-enhancer in HIV combination therapies. This was examined by performing EVG metabolic studies in human microsomes and drug quantification using liquid chromatography–tandem mass spectrometry (LC-MS/MS) method, CYP3A4 inhibition by EVG using Vivid^®^ assay, and EVG-CYP3A4 docking using molecular modeling.

## Material and Methods

### Chemicals and reagents

Elvitegravir, ritonavir, and cobicistat were purchased from Toronto Research Chemicals Inc. (Ontario, Canada). LC-MS grade acetonitrile, methanol, and formic acid were purchased from Sigma-Aldrich (Milwaukee, WI). An XTerra MS C18 Column was bought from the Waters Corporation (Milford, MA). Human liver microsomes (# HMMC-PL), pooled from 50 different individual donors to equally represent a truer population sample, was obtained from Invitrogen (Carlsbad, CA).

### Preparation of standards and quality control solutions

Analyte EVG and internal standard (IS) RTV stocks were prepared in methanol at 1.5 mg/ml. These stocks were further serial diluted in methanol to obtain 1–150 mg/ml stock concentrations. Working solutions were prepared by diluting the stocks in 0.1 M potassium phosphate buffer to maintain <1% of organic solvent concentration in the final reaction mixture in order to prevent the inactivation of microsomal enzymes. Series of ten microsomal calibration samples (0.003, 0.006, 0.013, 0.025, 0.050, 0.1, 0.15, 0.25, 0.5, and 1.0 μM) were made by adding appropriate volumes of microsomal suspension and working standards of EVG. Quality control (QC) solutions were prepared as the standards samples at three different concentrations (0.009, 0.2, 0.8 μM) to represent low, medium, and high QCs, respectively. The selection of calibration curve range was based on the sensitivity of the instrument and available data on EVG physiological concentration. The final concentration of IS in the standards, QCs, and experiments was 0.14 μM.

### LC MS/MS conditions

LC MS/MS system is consisted of Triple Quad 5500 from AB SCIEX (Framingham, MA), Shimadzu Nexera HPLC, and autosampler (Shimadzu Corp., Kyoto, Japan) controlled by Analyst^®^ software from AB SCIEX. The mass spectrometer was equipped with electron spray ionization (ESI) source in the positive mode. The best fragment ions for the EVG as well as IS were determined by optimizing compound dependent parameters. The multiple reactions monitoring (MRM) transitions (m/z) Q1/Q3 selected for quantitative analyses were 447.9/343.8 and 721.3/296.1 for EVG and RTV, respectively. Chromatographic separations were performed on an Xterra^®^ MS C18 Column (125Å, 3.5 μm, 4.6 mm X 50 mm). The following step-wise gradient was used to achieve the separation as well as to elute the analytes after the dead time (t_0_) of the column (0.58 minutes). Initially, both pump A (water with 0.1% formic acid) and pump B (acetonitrile with 0.1% formic acid) were at 50% for 1.50 minutes. Then, the concentration of pump B was gradually increased to 60% for 5.10 minutes to elute the analytes and to reduce the carry-over effect. The autosampler was maintained at 12°C throughout the run. The flow rate was kept at 1000 μl/min and the injection volume for each sample was 6 μl. A simple protein precipitation technique was used for extraction of the compounds. Microsomal samples were precipitated by adding acetonitrile (1:3 dilution) containing 0.14 μM final concentration of IS. Following vortex, the samples were centrifuged for 10 minutes at 8000 rpm. After centrifugation, supernatants were carefully collected without disturbing the pellet and transferred to glass inserts for the LC MS/MS analysis.

### Method validation

The developed method validation was performed by considering the guidelines published by FDA and previous literature [20, 21]. A ten-point calibration curve was generated and fitted to quadratic regression with 1/x^2^ weighting factor for the peak-area ratios (drug peak area/IS peak area) versus concentration. The weighting factor for the regression analysis was chosen based on a recently published report [22]. The specificity and selectivity of the method were examined by analyzing blank microsomal samples for the extracted lower limit of quantification (LLOQ) (0.003 μM). The percentage of signal to noise ratio from blank matrix analyses did not show significant impact on the analyte peak intensity (<15%).

Accuracy and precision of the method were determined by analyzing QC samples that cover the calibration curve range of the analyte. The low QC concentration was selected to be three times the LLOQ as per the FDA guidelines[20]. Inter-day precision and accuracy were calculated by analyzing extracted calibration standards and three QCs at different concentrations as described in our previous publications [13, 23]. The precision indicates the degree of variation and accuracy shows the bias between nominal and calculated concentrations. Carry-over (memory) effect was determined by assessing the extracted blank immediately after the upper limit of quantification (ULOQ, 1000 ng/ml). Matrix effect (ME), recovery efficiency (RE), and process efficiency (PE) were explored by following a previously proposed methodology [21]. The three sets of samples used for the experiments were as follows: A, pure standard solutions of EVG; B, blank microsomal extraction spiked with pure standard solutions of EVG; C, microsomal samples spiked with pure standard solutions of EVG and extracted at the low, medium, and high QC concentrations. After quantitation of the analyte the parameters were calculated using the following formulae. ME (%) = B/A x 100, RE (%) = C/B x 100, PE (%) = C/A x 100. Furthermore, analyte peak areas were corrected with their respective IS peak areas and reported. Finally, sample stability studies for the EVG in the microsomal matrix was tested using low, medium, and high QCs at the bench-top (25°C) and storage conditions (-80°C). Analyte concentrations in these samples were measured after fresh preparation and at 12, 24, and 48 hours. Stability of the EVG after undergoing freeze-thaw cycles was also examined up to three cycles in triplicates. For the long-term storage, samples were analyzed after storing them at the -80°C for three months. All determined concentrations were compared with freshly prepared QCs and reported the variation as a percentage of change from the freshly made QCs. The incurred sample reanalysis was also done by randomly selecting 10% of the total samples analyzed. The obtained data was compared with the first time results of the samples to determine whether the variability is beyond ± 20%.

### EVG metabolism in the human liver microsomes

The reaction was carried out using human liver microsomes in 0.1 M potassium phosphate buffer at pH 7.4 as described previously [23]. Briefly, the pre-incubation reaction mixture contains 10 mM MgCl_2_, 1 mg/ml microsomes, 0 or 20 mM ethanol, and varying concentrations (4, 8, 20, 40, 60, 80, 100, 120, 160, and 200 μM) of EVG and/or COBI in potassium phosphate buffer for substrate kinetic study. The same ratio of EVG and COBI is maintained to reflect Stribild^®^ formulations. After pre-incubation at 37°C for 10 minutes, 3 mM final concentration of NADPH was added to initiate the metabolic reaction. After 7 minutes incubation at 37°C the reactions were terminated by adding 1:3 volumes of cold acetonitrile. For the time kinetic study, the reaction was terminated at various time points (0.0, 2.5, 5, 10, 15, 20, 30, and 60 minutes). In this study, 4 μM EVG and either 4 μM (saturating concentration) or 0.04 μM COBI (sub-saturating concentration at its IC_50_ [24]) were used. The Prior to LCMS/MS analysis the analyte was extracted from the microsomes by a protein precipitation method as described previously [25].

### Inhibition of CYP3A4 activity by EVG

CYP3A4 inhibition assay was carried out by using Vivid^®^ CYP450 Screening Kit from Life Technologies as recommended by the manufacturer (# P2858, Carlsbad, CA). This assay allows rapid measurement of interactions between drugs of interest and CYP enzymes based on their capability to inhibit the production of a fluorescent signal in reactions using CYP BACULOSOMES^®^ Plus Reagents and specific Vivid^®^ Substrates. Briefly, the assay was conducted in a 96-well assay plate. Stock solution of EVG (100 mM) and COBI (12 mM) was prepared in acetonitrile. The final concentrations of 2.5, 5, 7.5, 10, 25, 50, 75, and 100 μM were achieved by diluting the stocks in 1X Vivid^®^ CYP reaction buffer. The same EVG concentrations were maintained for the experiments that use 0.04 μM of COBI). For ethanol only experiment, 2.5, 5, 10, 10, 20, 30, and 50 mM final concentrations were used. The final concentration of acetonitrile in each reaction was 0.01% in 100 μl final reaction volume. The fluorescence was immediately measured using a microplate reader (Cytation™ 5 Cell Imaging Multi-Mode Reader, BioTek, VT) at the 415/460 nm excitation and emission wavelengths.

### Molecular docking of EVG, COBI to CYP3A4 model

The initial CYP3A4 model for docking was taken from Protein Data Bank (PDB) [26]. 3NXU crystal structure was chosen for docking based on the completeness, resolution, and ligand size. The CYP3A4 model was subjected to energy minimization by the protein preparation wizard module in Schrödinger [Schrödinger, LLC, New York, NY, 2012]. The coordinates of EVG, COBI were extracted from PDB. The ethanol was built in Maestro. All ligands were optimized by the Epik [27] module in Schrödinger. During the dockings, the ligands were treated as full flexibility, while a static model was used for the protein due to its large active site. All docking simulations were accomplished by GOLD suite 5.2 [CCDC Software Ltd., Cambridge, UK]. Residues within 20 Å of the ligand were defined as the binding pocket. Chemscore was used for scoring the ligand-CYP3A4 interactions. Number of output solutions was set to 100 for each run. The first 10 poses were analyzed in detail.

### Data analyses

The concentrations of all the drugs were quantified using Ab Sciex Analyst^®^ software. Microsomal kinetic data were analyzed using Michaelis-Menten and exponential decay equations. CYP3A4 inhibition data were evaluated by fitting the values to dose-response inhibition and calculated IC_50_ values were reported. All graphs were plotted and two-tailed t-tests were performed wherever appropriate using GraphPad Prism 5.01 (GraphPad, San Diego, CA). Significant differences between more than two groups analyzed with one-way ANOVA followed by Dunnett’s multiple comparison tests using IBM SPSS Statis41tics version 20. A p value <0.05 was considered significant compared to respective controls. The original data and/or statistical analyses for Michaelis-Menten Kinetics, drug metabolism and half-life determination, inhibition studies, and ligand docking are provided in the supporting information (S1 file).

## Results

### Method development

**Fig 1:**
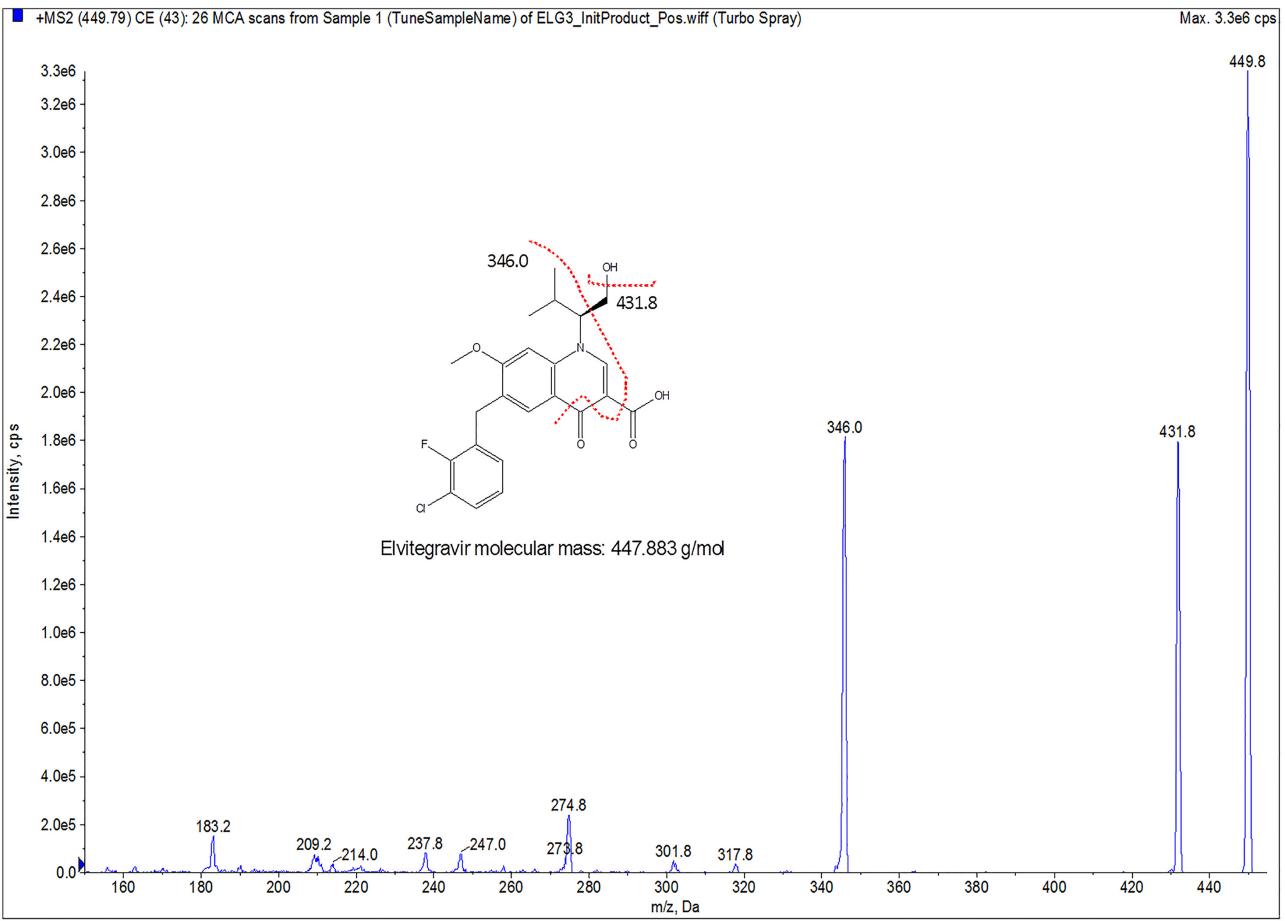
MS/MS spectrum of elvitegravir (EVG) with proton adducts [M+H]^+^ in ESI-positive mode. The y-axis shows the intensity (CPS, count per second); the x-axis shows the mass to charge ratio (*m/z*,*Da*). The EVG structure was made using ChemDraw Ultra (version 6.0.1; CambridgeSoft.com). Red dotted lines on the structure indicates the fragmentation pattern of EVG.

Pre-collision cell voltages such as declustering potential (DP) and entrance potential (EP) were at 100 and 10 V, respectively, for the analyte EVG as well as IS RTV. After determining the maximum intensity precursor ion with proton adducts [M+H]^+^ for EVG and RTV, the collision energy (CE) and collision cell exit potential (CXP) for each product ion were optimized. The optimal CE and CXP values for EVG were 43 and 20, respectively, while these values for RTV were 27 and 29, respectively. The other parameters used in this method development are: Curtain Gas (CUR)– 20, IonSpray Voltage (IS)– 5500, Temperature (TEM)– 500°C, Ion Source Gas 1 (GS1)– 50, Ion Source Gas 2 (GS2)– 50, collision-activated dissociation (CAD) gas– 8. A representative MS/MS spectrum for MRM transitions of Q1 and Q3 for quantitative analysis of EVG is shown in Fig 1.

**Fig 2:**
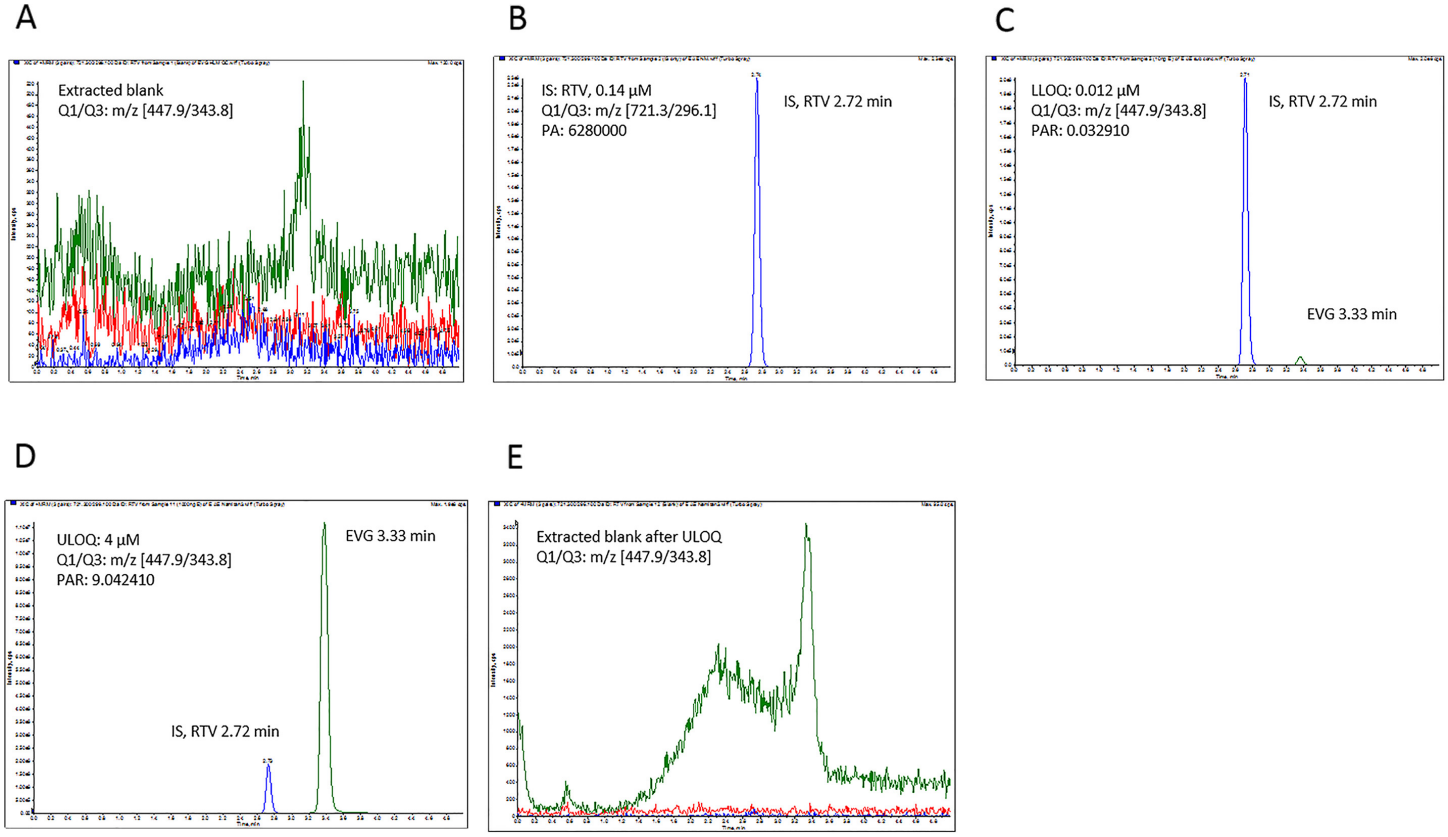
Multiple reaction monitoring (MRM) chromatogram peaks of elvitegravir (EVG) and its corresponding internal standard (IS), ritonavir (RTV). (A) Extracted blank (y axis:0–520 CPS), (B) extracted IS (0.14 μM; y axis:0–2.3e^6^ CPS), (C) extracted lower limit of quantitation (LLOQ, 0.003 μM; y axis:0–2.0e^6^ CPS), (D) upper limit of quantitation (ULOQ, 1 μM; y axis:0–1.10e^7^ CPS), (E) blank after ULOQ (y axis:0–3400 CPS). The y-axis shows the intensity (CPS, count per second); the x-axis indicates the run time in minutes. PA, peak area: PAR, peak area ratio (analyte/IS).

Fig 2C and 2D show representative chromatograms of EVG samples at 0.012 and 4 μM, respectively, using LC conditions and positive ESI conditions as described in the material and methods section. The retention times for EVG and RTV are 3.33 and 2.72 minutes, respectively. Although both EVG and IS are eluted in less than 4 minutes total run, the run time was extended up to 5 minutes to increase the column washing time.

### Method validation

The concentration (0.003–1.0 μM) range for calibration curve was chosen based on instrument sensitivity as well as required detection range for our experiments (Table 1). The representative chromatographic profile of EVG and IS in the microsomal matrix were shown in Fig 2. The signal-to-noise ratio of extracted blank was <3% of EVG at the mean peak area ratio at the LLOQ and ULOQ with the IS (Fig 2A, 2C and 2D). The extracted IS peak area (0.14 μM) is shown in Fig 2B. The levels of quality control (QC) samples 0.009, 0.200, and 0.800 μM were selected to reflect low, medium, and high range of calibration standards (Table 1).

**Table 1:** Inter-day accuracy and precision of calibration standards (n>6) and quality control (QC) standards (n>6) of elvitegravir in human liver microsomes.

Name	Calibration curve standards										Quality Control		
	1	2	3	4	5	6	7	8	9	10	Low	Medium	High
**Nominal conc. (μM)**	0.003	0.006	0.013	0.025	0.050	0.100	0.150	0.250	0.500	1.00	0.009	0.200	0.800
**Conc. found (μM)**	0.0027	0.0067	0.0135	0.0306	0.0533	0.1011	0.1668	0.2526	0.5042	1.0477	0.0091	0.2010	0.7840
**Precision (CV) %**	7.09	14.43	11.34	14.48	11.98	3.45	12.19	5.32	4.97	11.90	3.11	7.16	5.32
**Accuracy (Bias) %**	109.09	89.47	95.96	85.24	93.85	98.90	89.94	99.00	99.19	95.47	106.38	103.06	98.51

CV: coefficient of variation (precision)

Precision and accuracy were measured using calibration curve standards as well as at low, middle, and high QC concentrations as presented in Table 1. The mean inter-day precision is acceptable with coefficient of variation (CV) at <15%. The inter-day bias (deviation) from the nominal concentrations of calibration standards as well QCs varied from 0.8% to 15%. The EVG signal detected in the microsomal blank after the ULOQ is ±0.1%, which corresponds to ±4% of LLOQ value for EVG (Fig 2E). Overall, the selected calibration curve range, accuracy, and precision of LLOQ are less than 20% limit as recommended by FDA bioanalytical method validation guidelines [3].

Matrix effect (ME), recovery (RE), and process efficiency (PE) are reported using low, medium, and high QC concentrations (Table 2). For ME, the variation above or below 100% reflects ionization enhancement or suppression, respectively. With increasing concentration, slight ionization suppression was observed for EVG (B/A ratio). However, this suppression effect was nullified when normalized with IS (B2/A2 ratio). Similarly, calculated RE and PE values are satisfactory and within the recommended limits.

**Table 2:** Matrix effect (ME), recovery (RE) and process efficiency (PE) of elvitegravir (EVG) (n = 6).

Nominal conc.(μM)		ME (%)				RE (%)				PE (%)			
		B/A	CV	B2/A2^a^	CV	C/B	CV	C2/B2 ^a^	CV	C/A	CV	C2/A2 ^a^	CV
**LQC**	**0.040**	100.78	3.5	106.34	1.70	104.92	1.7	99.11	4.8	105.74	1.7	105.39	4.8
**MQC**	**0.75**	94.35	4.0	101.88	3.14	120.34	10.6	111.17	13.0	113.55	10.6	113.26	13.0
**HQC**	**3.50**	94.81	6.7	107.11	2.55	101.14	6.6	90.55	2.7	95.89	6.6	96.99	2.7

A: pure standard solutions of EVG; B: blank microsomal extraction spiked with pure standard solutions of EVG; C: microsomal samples spiked with pure standard solutions of EVG and extracted;

^a^ analyte/internal standard peak-area ratio; CV: coefficient of variation (precision); HQC, high QC; MQC, middle QC; LQC, low QC.

Stability data of EVG in the microsomal matrix is reported in Tables 3 and 4. The deviation of the analyte concentration over time and after freeze-thaw cycles are expressed as percentage of freshly made and analyzed QCs, respectively. Stability of the EVG at room temperature over 48 hours is considered stable as the variation is ±15% (Table 3). However, comparing ±15% variation of low end with the ±2% change at the higher end suggests that loss of analyte is much more significant at the lower concentrations compared to the high end one in the method. Moreover, percentage of variation in the freeze-thaw stability studies is less than 15%, suggesting no significant breakdown of the compound up to 3–4 freeze-thaw cycles (Table 4). Of note, EVG samples stored at -80°C for long term storage (3–4 months) also did not exhibit significant loss in the concentration (data was not shown).

**Table 3:** Time-dependent stability studies of elvitegravir at low (0.040 μM), medium (0.75 μM) and high (3.50 μM) quality control concentrations in triplicates. The variations are expressed as a percentage change from freshly prepared quality controls.

Stability(hours)	At Room Temp (25°C)		
	Low	Medium	High
0	0	0	0
12	-14	9	0.1
24	-10	4	1.8
48	-5	7	-0.4

**Table 4:** Freeze-thaw stability data of elvitegravir at low (0.040 μM), medium (0.75 μM) and high (3.50 μM) quality control concentrations in triplicates. The variations are expressed as a percentage change from freshly prepared quality controls.

Freeze-thaw Cycles	Freeze thaw (-80°C)		
	Low	Medium	High
1	-12	5	-3
2	-7	3	3
3	-3	5	-15

### Effect of ethanol on the EVG and EVG + COBI time-dependent metabolism in human liver microsomes

**Fig 3:**
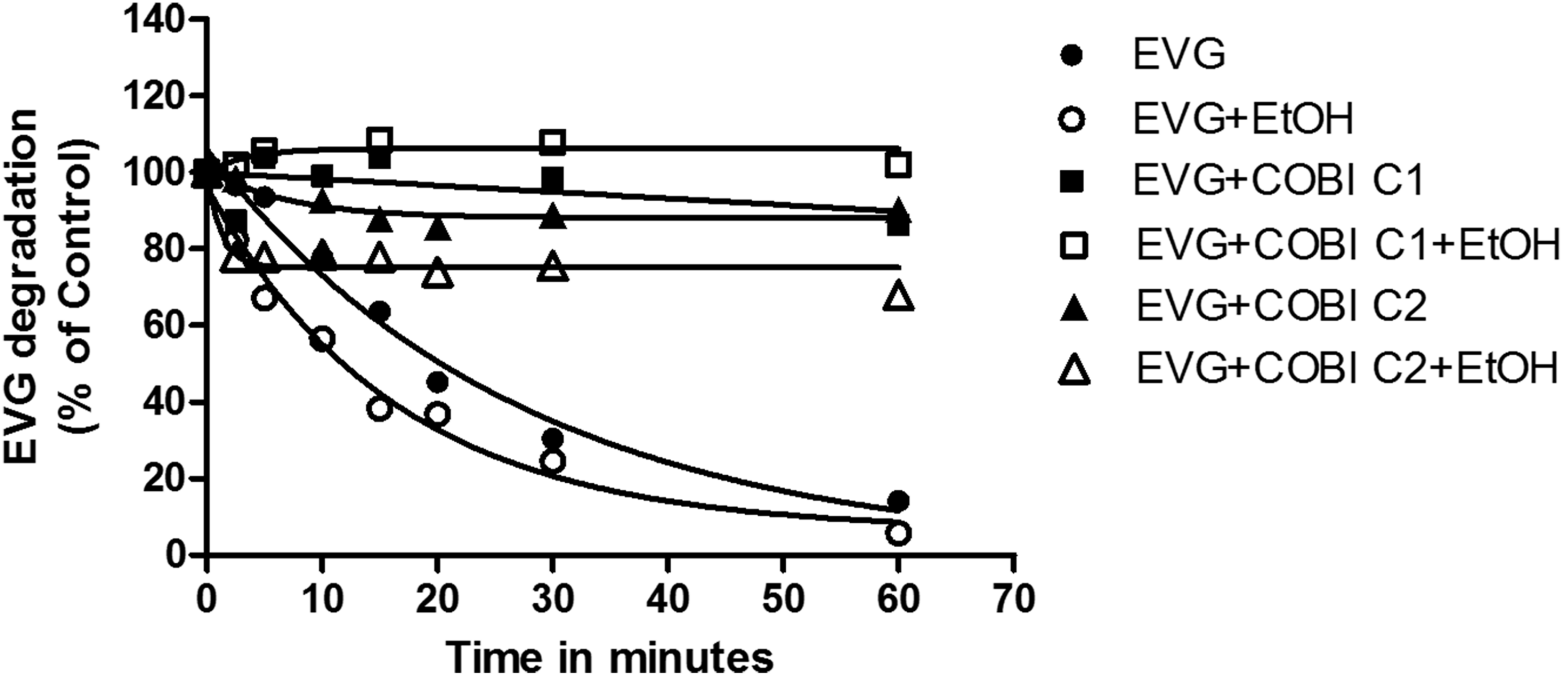
Kinetics of degradation of elvitegravir (EVG) in the human liver microsomes (HLM) in the absence and presence of 20 mM ethanol. The degradation of EVG (% of control) was plotted by measuring remaining substrate at the specified time intervals. Assay was performed using HLM as a source of enzyme as described in the materials and methods. Mean ± S.E.M values were calculated from the fitting of the curve. The results are representative of at least three independent experiments. COBI C1 represents 4 μM and COBI C2 represents 0.04 μM.

To study the influence of alcohol consumption on EVG metabolism, 20 mM ethanol (physiological concentration in moderate drinker) was used to treat human liver microsomes. Kinetic analysis of EVG metabolism in microsomes was performed in the presence of 20 mM ethanol and/or similar concentration of COBI and the results are presented in Fig 3 and Table 5. EVG degradation/metabolism at 4 μM followed a pseudo-first-order kinetic. Therefore, we fit the data to an exponential one phase decay equation to determine the apparent half-life (t_1/2_) and rate constant K.

**Table 5:** Kinetic Characterization of elvitegravir (EVG) in the presence and absence of cobicistat (COBI) and/or ethanol (EtOH)

Parameters	EVG	EVG+EtOH	EVG+COBI	EVG+COBI+EtOH	EVG +0.04 μM COBI	EVG + 0.04 μM COBI+ EtOH
**Half-life (minutes)**	17 ± 1.0	12 ± 0.6 *	7.4e+006 ± 1.874e+006 ^#^	2.53 ± 0.6 *^	6.4 ± 1.08	0.82 ± 0.03 *^
**K**	0.08 ± 0.04	0.05 ± 0.01	0.11 ± 0.04	0.16 ± 0.04	0.12 ± 0.02	0.07 ± 0.01*
**V**_**max**_ **(μM/min/mg/protein)**	162 ± 16	221 ± 18*	308 ± 45 #	2352 ± 467*	ND	ND
**K**_**m**_ **(μM)**	68 ± 17	115 ± 18*	189 ± 46 #	1653 ± 357*	ND	ND
**V**_**max**_**/K**_**m**_	2.4	1.9	1.6	1.4	ND	ND
**IC**_**50**_ **(μM)**	13.07 ± 1.1	17.54 ± 1.5	0.0002 ± 0.0 ^#^	0.0005 ± 0.0 *^	30.97 ± 2 ^#^	117.4 ± 3 *^

ND = not determined;

*p<0.05 compared to respective no ethanol treatment;

^#^p<0.05 compared to EVG alone;

^<0.05 compared to EVG+EtOH

In this study, saturating concentration of COBI (4 μM; dubbed as COBI C1) was used to match with the EVG concentration and reflect Stribild^®^ formulation. However, the obtained data did not fit well with the model. Therefore, we also used a sub-saturating concentration of COBI at its reported IC_50_ (0.04 μM; dubbed as COBI C2) [24], while keeping EVG concentration constant. The t_1/2_ for the EVG metabolism in the presence of ethanol was decreased by about 30% compared to EVG alone (12 vs. 17 minutes, p<0.05 student t test), which is also apparent from a decrease in the rate constant by similar magnitude (Table 5). The results showed that ethanol exposure significantly decreases the EVG half-life (p<0.05 student t test, Fig 3, Table 5). As expected, EVG+COBI C1 half-life was increased significantly compared to EVG (F_(2,8)_ = 47, P = 0.002, one-way ANOVA). Ethanol presence in both COBI conditions elevated the half-life values significantly compared to EVG+EtOH treatment (F_(2,8)_ = 1249, P<0.001, one-way ANOVA). Even though there was a decreasing trend in rate constants, we did not observe statistically significant difference in multiple comparisons. As depicted in Fig 3, some of the EVG+COBI combinations did not exhibit any EVG degradation. However, in figure it appears that they are slightly above the control value, which is due to 10–15% variability when quantitating the EVG in LC-MS/MS. Overall, these data suggest that the 20 mM ethanol may increase the rate of EVG metabolism at its physiological concentrations in the microsomes.

**Fig 4:**
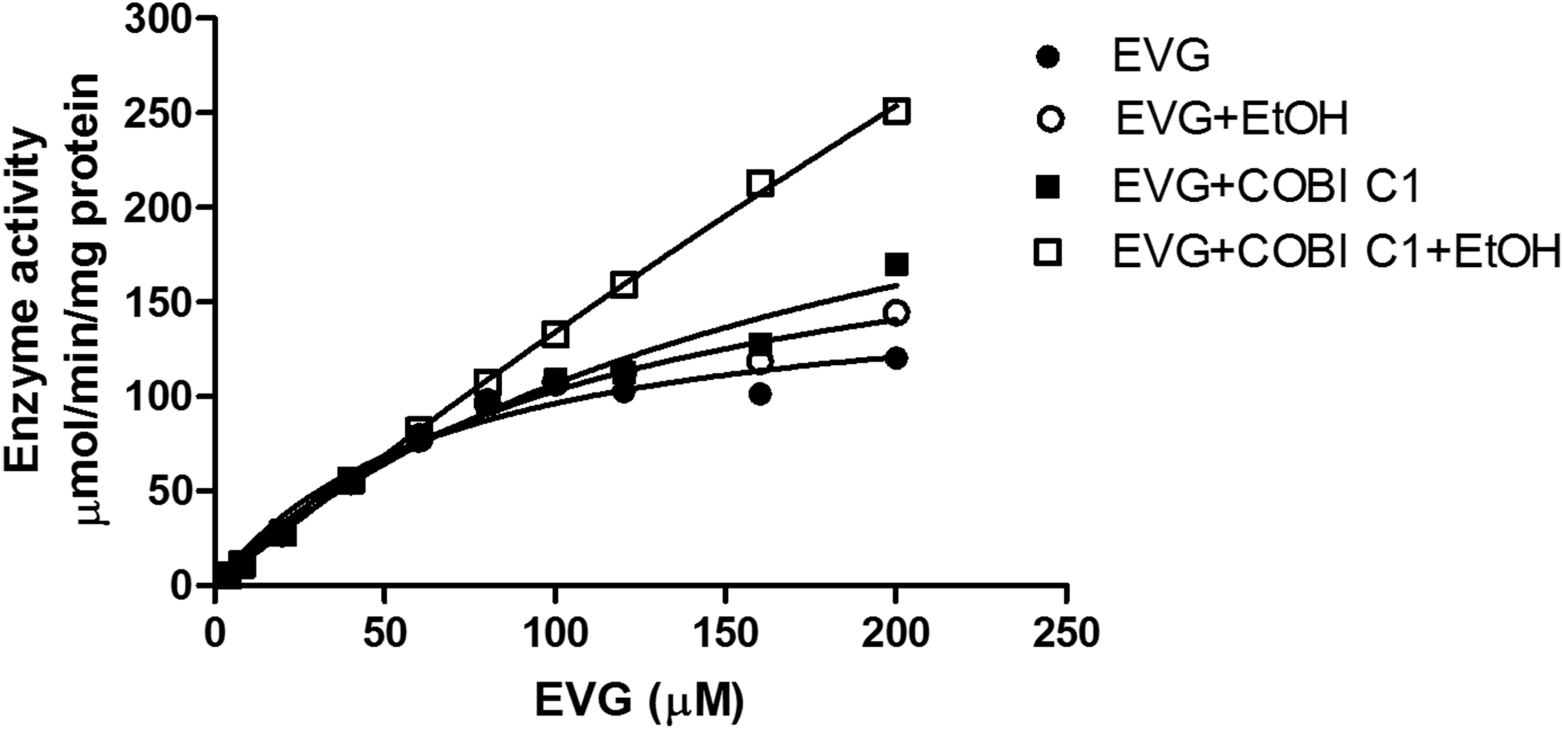
Saturation kinetics of elvitegravir (EVG) in the human liver microsomes (HLM) in the absence and presence of 20 mM ethanol. The maximum enzyme velocity (V_max_) and Michaelis-Menten constant (K_m_) were calculated by fitting the data to Michaelis-Menten model and enzyme activity was reported as μmol/min/mg protein. Mean ± S.E.M values were calculated from the fitting of the curve. The results are representative of at least three independent experiments.

In the presence of ethanol both the V_max_ and K_*m*_ values were increased significantly compared to EVG alone (V_max_ = 162 ± 16 vs. 221 ± 18 μmol/min/mg protein, and K_m_ = 68 ± 17 vs. 115 ± 18 μM; p<0.05 student t test, Table 5). Similarly, when COBI is present both the V_max_ and K_*m*_ values were increased compared to EVG alone (V_max_ = 162 ± 16 vs. 308 ± 45 μmol/min/mg protein, and K_m_ = 68 ± 17 vs. 189 ± 46 μM). However, in the presence of COBI and ethanol together the substrate kinetics did not saturate and the Michaelis-Menten kinetics yielded very high mathematical values for V_max_ and K_m_ (Fig 4 and Table 5). Even though these values are statistically significant they are not biologically relevant. Importantly, ethanol or COBI alone exhibited a decreasing trend of enzyme efficiency (V_max_/K_m_) compared to EVG alone.

### Inhibition of CYP3A4 by EVG, COBI and/or ethanol

**Fig 5:**
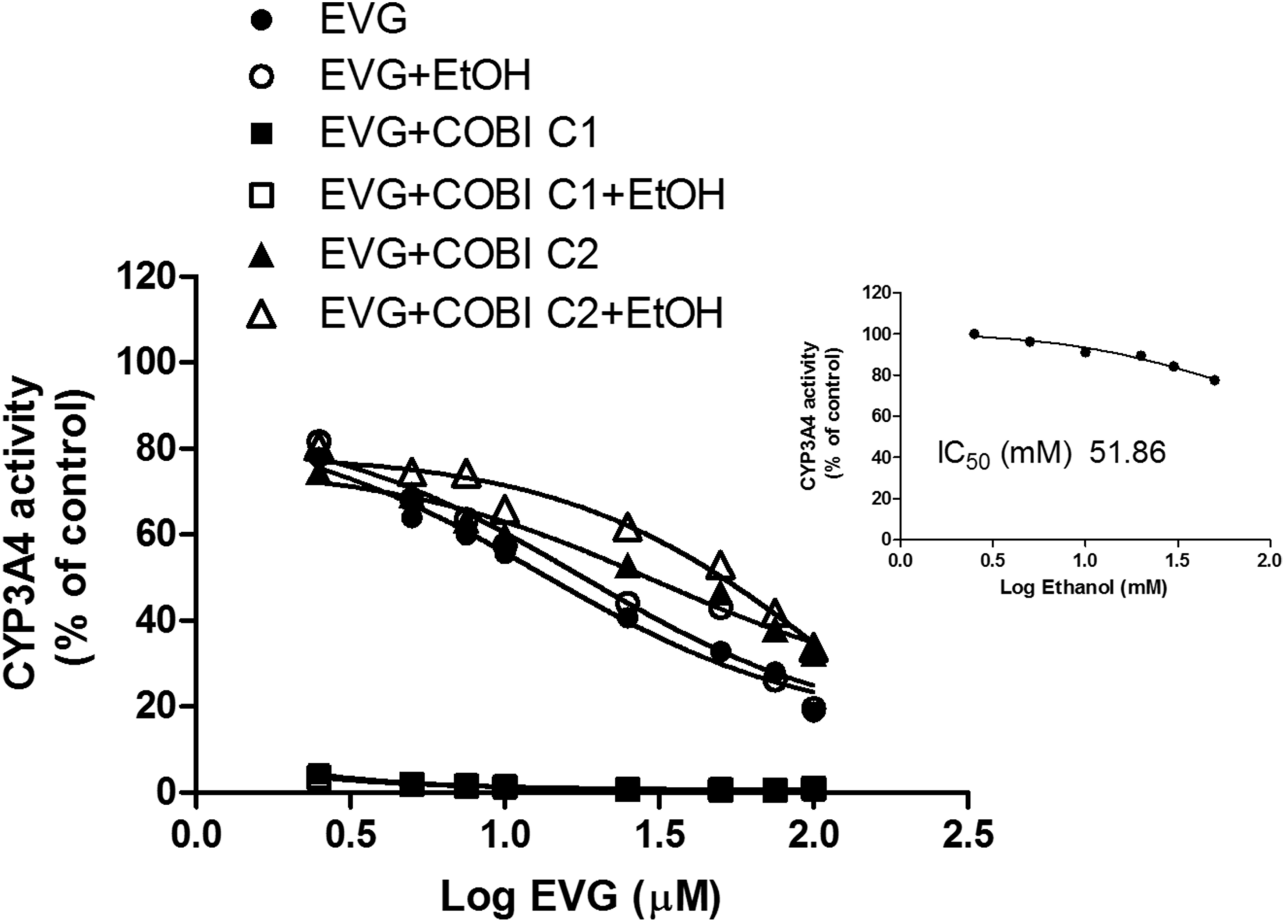
Inhibition of human recombinant CYP3A4 activity by elvitegravir (EVG) and/or cobicistat (COBI) in the presence and absence of 20 mM ethanol. Enzyme activity was determined by using vivid^®^ blue screening kit with baculosomes containing CYP3A4. Dose-response inhibition curve generated by plotting log EVG concentration on x-axis and CYP3A4 activity on y-axis. Data are expressed as the mean ± S.E.M. The results are representative of at least three independent experiments. The CYP3A4 inhibition by ethanol alone is presented in the inset. COBI C1 represents 4 μM and COBI C2 represents 0.04 μM.

The inhibition of CYP3A4 enzyme activity by EVG and/or COBI in the absence and presence of ethanol was measured using vivid^®^ blue screening kit and the results are presented in Fig 5 and Table 5. As a control, ethanol only treatment on CYP3A4 showed an IC_50_ value of 51.86 mM, and ~10% inhibition at 20 mM (Fig 5 inset), which was used to study the effect of ethanol on CYP3A4 inhibition by EVG. EVG showed an IC_50_ of 13.07 ± 1.1 μM for CY3A4 inhibition. Ethanol at 20 mM exhibited 34% increase in the IC_50_ compared to EVG alone (17.54 ± 1.5 vs. 13.07 ± 1.1 μM). We also used 10 and 50 mM ethanol to determine if below or above physiological ethanol concentration show different effect on the IC_50_ of EVG-CYP3A4 inhibition compared with physiological concentrations. These results also exhibited similar decrease in the IC_50_ values. EVG+COBI at the matching concentrations showed a robust decrease in overall IC_50_ values (Fig 5). However, ethanol significantly increased the IC_50_ of EVG+COBI (0.2 vs. 0.5 nM. P<0.05, student t test). Since there was a robust decrease in the IC_50_ value at 1:1 ratio of EVG to COBI, we performed experiment at varying EVG concentrations and 0.04 μM COBI (IC_50_ of COBI). The results showed a significant increase in the IC_50_ of EVG+COBI at sub saturating COBI concentration compared with EVG alone and EVG+COBI at 1:1 ratio (F_(2,8)_ = 435, P<0.001, one-way ANOVA). Furthermore, ethanol also increased the IC_50_ of EVG+COBI at sub-saturating COBI concentration (F_(2,8)_ = 5112, P<0.0001, one-way ANOVA).

### Docking of EVG and COBI to the human CYP3A4 in the presence and absence of ethanol

**Fig 6:**
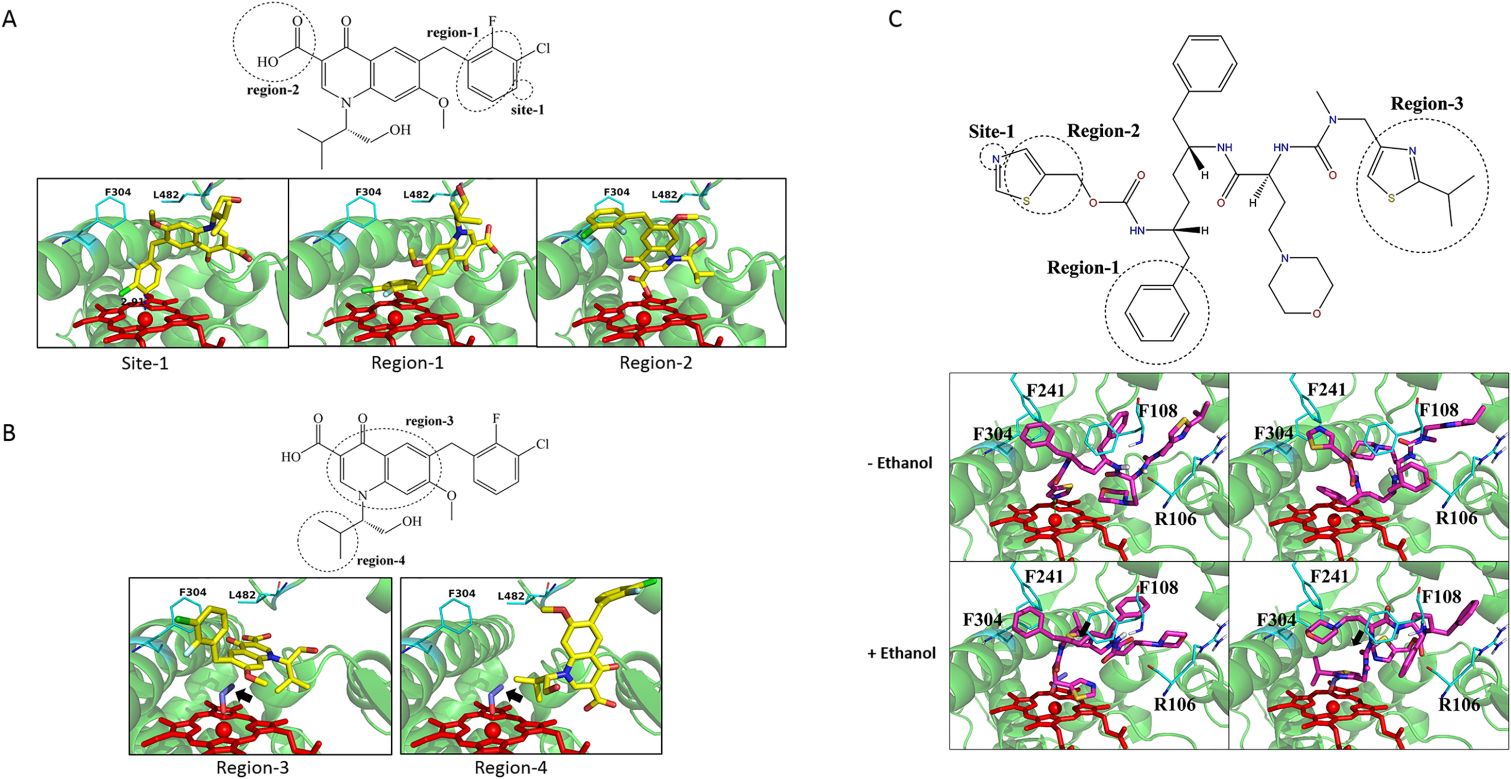
Docking of elvitegravir (EVG), cobicistat (COBI) with the human CYP3A4. EVG docking in the (A) absence and (B) presence of ethanol. (C) Docking of COBI in the absence and presence of ethanol. Chemscore was used for scoring the ligand-CYP3A4 interactions. The simulated interactions are grouped into clusters: site-1, region 1–4 (see text for the details). CYP3A4 is shown in light green, EVG in yellow, COBI in magenta, heme of the P450 in red and alcohol in indigo. Black arrows show ethanol molecule.

To further confirm the influence of ethanol on EVG-CYP interactions, molecular docking with CYP3A4 (the major EVG-metabolizing CYP) was performed using GOLD suite 5.2. Based on the substrate docking scores the first 10 poses in the absence of ethanol were categorized into 3 clusters A: Site 1 (site of metabolism) points to the heme iron. B: Region-1 points to the heme. C: Region-2 points to the heme (Fig 6A and Table 6). Likewise, the first 10 poses in the presence of ethanol can be catalogued as 2 primary clusters. A: Region-3 points to the heme. B: Region-4 points to the heme (Fig 6B and Table 6). In general, docking in the presence of ethanol showed alterations in the overall average docking scores of top 10 poses. In addition, the average distance and orientation towards the metabolic site was changed compared to EVG docking alone. Taken together, these results suggest that ethanol does alter the binding pattern of EVG with CYP3A4 active site.

**Table 6:** The statistical results of elvitegravir (EVG), cobicistat (COBI) docked into the CYP3A4 active site in the absence and presence of ethanol (EtOH).

EVG	Num	Average score	Highest score	Average distance (A)^[a]^
Site-1	4	37.10	38.0	2.96
Region-1	4	36.51	37.30	3.11
Region-2	2	37.84	38.53	2.56
**EVG + EtOH**				
Region-3	6	37.64	39.91	6.09
Region-4	3	34.76	34.92	5.65
Other	1	35.80	35.80	5.64
**COBI**				
Site-1	8	60.60	65.30	2.60
Region-1	2	59.3	63.2	3.05
**COBI + EtOH**				
Region-3	2	50.60	52.10	5.57
Region-4	4	50.20	51.50	5.51
Other	4	49.30	51.50	5.62
**EVG + COBI**	not determined			
**EVG + COBI + EtOH**	not determined			

^[a]^The averaged distance between the atom of the site closest to Fe

Ligand docking was also performed with COBI at the active site of CYP3A4 at the absence and presence of ethanol. The top 10 poses of docking in the absence of ethanol were grouped into 2 clusters with Site-1 or Region-1 pointing to heme (Table 6 and Fig 5C). As shown in Table 6, Site-1 was the major cluster with the highest docking score of 65.3, which was much higher than that of EVG (38.0; Table 6). The N atom of the thiazole group was ligated to the heme iron with an average distance of 2.6 Å. With ethanol in the binding site, the cluster numbers in the top 10 conformers became a little complicated. The average distances between the closest atom and Fe in the top 10 conformers were greater than 5Å. Docking scores of these conformers significantly decreased to ~50 when compared to those without ethanol bound. Finally, we attempted to dock EVG in the presence of COBI, and COBI + ethanol at the active site of CYP3A4. However, due to larger sizes of EVG and COBI together they failed to dock.

## Discussion

In the present study, we investigated the effect of ethanol and/or COBI on the metabolism of EVG in human liver microsomes, as well as, their effects on the inhibitory influence of EVG on CYP3A4. Initially, we developed and validated a simple and sensitive LC-MS/MS method for the simultaneous quantification of antiretroviral drugs EVG and RTV followed by characterization of the kinetics of EVG metabolism in microsomes. Overall our results suggest that ethanol at physiological concentration alters EVG metabolism, as well as, the ability of EVG to inhibit CYP3A4 (major EVG-metabolizing CYP enzyme), which can also be explained by molecular docking. However, our study could not fully explain the effects of ethanol in EVG and COBI co-administered conditions. This is the first study reporting the effect of ethanol on EVG metabolism and inhibition of CYP3A4 by EVG in the absence and presence of ethanol and/or COBI.

The proposed ESI LC-MS/MS method was developed by considering various factors that include sample preparation and extraction, mass spectrometry parameters, and chromatographic conditions that dictate the quality and reliability of the process [28]. Sample preparation is one of the key aspects for the success of any bioanalysis. Protein precipitation approach with acetonitrile produced good extraction efficiency with less matrix effect (Table 2). A slight ion enhancement effect that was observed in the extracted EVG samples may be due to the presence of endogenous compounds such as sulfates, phosphates, and sodium from the microsomes [29]. This effect may be eliminated or reduced to minimum level by using other effective strategies like liquid-liquid extraction and solid-phase extraction, however, these methods are labor-intensive, expensive, and time consuming.

To develop a rapid, robust, and sensitive method, we tested mobile phase consisting different ratios of acetonitrile, water, and formic acid. Initial mobile phase with isocratic flow (acetonitrile: water: formic acid: 60:30:0.01) eluted the drugs RTV and EVG at 1.31 and 1.99 minutes, respectively, which is considered as a rapid method. But the caveats are that the analytes do not have sufficient time to interact with the column material and they elute as such without proper separation. Moreover, eluting the analytes within the void volume range increases the ion suppression effects in the LC-MS/MS [30]. These issues were resolved by opting a gradient mobile phase and increasing the injection flow rate. These modified parameters improved the peak shapes and shifted the analytes RTV and EVG peaks location to 2.7 and 3.3 minutes, respectively. Carry-over or memory effect is one the major concerns in a bioanalytical method development. EVG showed more carry-over effect than the IS RTV. There are several factors that contribute to the carry-over effect, especially sample preparation, column, and autosampler. Column carry-over is very compound-dependent and this effect can be minimized by choosing gradient elution, increasing washing, or altering the chromatographic run time [31]. One extra minute of washing time was introduced at the end of all the analytes elution to reduce the memory effect from the column, because it was observed that EVG had strong tendency to interact with reverse phase C18 column material. This problem was also addressed by optimizing the autosampler rinsing volume, speed, and mode since autosampler carry-over is a persistent problem that can compromise accuracy and precision of the analyte, especially at the lower concentrations [32].

LC-MS/MS is the method of choice for the quantitative determination of the drugs in the complex biological matrices. However, endogenous impurities from biological samples may interfere with the selectivity of the analyte signal. Therefore, we investigated the ME, RE, and PE by following an experimental protocol proposed previously [21] (Table 2). Comparing the MS/MS response to blank microsomal extraction spiked with pure EVG to MS/MS response of pure EVG in mobile phase provides the absolute matrix effect. EVG showed a little ion suppression effect as the concentration of the drug increases in the reaction mixture. This effect is in agreement with a recent report that determined EVG concentrations in plasma samples of HIV positive patients [33]. Even though precision values are within the limits of recommendation, it appears that overall percentage of change in the RE and PE values are >100%, especially at low and medium concentrations indicating a possible ion enhancement effect in the MS/MS response. This may due to the ME, which is more pronounced in the mass spectrometers with ESI ion source than atmospheric pressure chemical ionization (APCI) [34]. Thus, the developed method is simple, robust, and selective for the quantitation of new generation antiretroviral drug EVG in the human liver microsomal matrix.

EVG is primarily metabolized via hepatic CYP3A4 enzyme-mediated aromatic and aliphatic hydroxylation and glucuronidation by phase II enzyme. CYP3A4 and glucuronidation pathways generate metabolites GS-9202 (M1) and GS-9200 (M4), respectively [6]. In human liver microsomes EVG metabolism via CYP3A4 is more extensive than glucuronide conjugation. Plasma pharmacokinetics reported half-life values for EVG and COBI-boosted EVG dosing, as a result of mainly liver CYP3A4-mediated metabolism, were 2-3hr and 8hrs respectively [3]. In this microsomal study, COBI at saturating, but not sub-saturating concentrations, greatly increased the apparent EVG half-life. Perhaps, EVG dominantly interact with CYP3A4 at sub-saturating concentration of COBI. However, this interaction is reduced at high COBI concentration leading to decreased metabolism of EVG. It is not appropriate to compare the reported half-life values obtained from the plasma with our results, which is based on apparent half-life of EVG from microsomal CYP3A4-mediated metabolism. Nonetheless, irrespective of the concentration of the COBI, existence of ethanol in the sample clearly reduced EVG half-life and rate constant, suggesting that ethanol induced decrease in EVG interaction with CYP3A4.

Based on the EVG metabolic activity the K_m_ values were reported to be 21.46 μM [3]. The K_m_ for EVG metabolism by CYP3A4 in this study is relatively higher than the reported value (68 μM, Table 5). This discrepancy could be due to many factors such as source of microsomes, quantification of EVG, and the experimental design for the assay. At the physiological concentration of EVG and COBI, COBI considerably inhibited the enzyme activity and therefore obtained V_max_ and K_m_ values were relatively high from the EVG only samples. Ethanol exposure showed a statistically significant effect on EVG+COBI combination but it is not sufficient to draw affirmative conclusions from this data. However, at multiple concentrations of EVG, which are much higher than physiological concentration, we were able to obtain the effect of COBI on enzyme efficiency of CYP3A4 for EVG metabolism. Although it may not have physiological significance COBI clearly increased both V_max_ and K_m_ with overall decrease in enzyme efficiency.

ART drugs are often involved in drug-drug interactions with CYP3A4 enzyme, specifically protease inhibitors and NNRTIs. Most of these drugs are not only substrates for CYP3A4 but can also act as inhibitors as well as inducers [35]. Consistent with the literatures EVG is also a weak inhibitor of CYP3A with the IC_50_ value of 13 μM (Fig 4, Table 5). This inhibitory effect appear to be insignificant when compared with CYP3A4 inhibition by protease inhibitors. For example, ritonavir, indinavir, and saquinavir inhibit CYP3A4 at the IC_50_ values of 0.034, 0.43, and 2.14 μM [36]. However, as EVG is co-administered with other antiretroviral drugs and pharmacoenhancer such as COBI or RTV, it is expected that these combination therapies may alter the inhibitory effects of EVG on the CYP3A4 enzyme [1] at least during the acute phase of drug exposure. Therefore, we tested for this possibility in the presence of EVG matched COBI concentration (recommended dose in clinical practice) as well as 0.04 μM (COBI concentration at its reported IC_50_ value) (Table 5). The affinity for CYP3A4 interactions with EVG and COBI at high COBI concentrations was much higher than the EVG alone. However, the affinity for this interaction was reduced when COBI was present at sub-saturating concentration. The presence of ethanol in these samples significantly reduced the affinity of EVG for CYP3A4 (enhanced IC_50_ values) regardless of COBI concentration. These results implies that there is a possibility for ethanol-EVG-CYP3A4 interaction with/without COBI, which may influence the way EVG binds to the enzyme. As IC_50_ values estimated in in vitro studies are relatively equivalent to the steady-state plasma concentrations of the drugs [5], it can be assumed that there may be a potential drug-drug interaction. Nevertheless, more conclusive evidence is necessary to support these kinetic properties of CYP3A enzymes for EVG drug metabolism.

Alcohol consumption is prevalent in more than 50% of HIV positive individuals as opposed to 20% individuals in normal population [37]. Furthermore, alcohol is known to induce HIV replication and decrease the efficacy of ART regimen [38]. To study the clinical relevance of the effect of alcohol on HIV patients, we have demonstrated that ethanol exposure differentially alters the binding and inhibition of protease inhibitors to CYP3A4, which in turn, affect the metabolism of these drugs [12, 13]. Kinetic results revealed that there was a decrease in the enzyme efficiency and nearly 30 percent decrease in the half-life (Figs 3 and 4, Table 5) for the metabolism of EVG in the presence of physiological concentration of ethanol. This clearly suggests a decrease in EVG metabolism at low physiological concentration (4 μM), but may not have significant effect at non-physiological higher concentration (equivalent to K_m_ value) of EVG. This outcome is similar to the previous finding of decreased efficiency of CYP3A4 for the metabolism of antiretroviral drug nelfinavir [13]. Similarly, an increase in IC_50_ in the presence of ethanol (Table 5) suggests decrease in binding affinity of EVG with CYP3A4 in the orientation that lead to CYP3A4 inhibition. In fact, our result did show a decrease in metabolic activity at low EVG concentration. Similar to these findings we have previously reported that ethanol significantly alters the IC_50_ for the protease inhibitors indinavir and ritonavir by perhaps facilitating the CYP3A4 and protease inhibitors interaction through hydrogen bonding and hydrophobic interactions [39].

Finally, these experimental findings were supported by our molecular docking studies. Existence of ethanol in the binding pocket changes the binding modes of EVG by altering the orientation of binding sites, as well as, it pushes the EVG binding regions far away from the heme iron (Fig 6B and Table 6). It can be speculated that the altered binding orientation by ethanol would lead to formation of new metabolites in the presence of ethanol, which could have a physiological impact. COBI being a stronger inhibitor of CYP3A4 than EVG also showed better scores and shorter distances with the heme compared to EVG. However, the average virtual distance of COBI towards CYP3A4 has been altered in the presence of ethanol (Fig 6C and Table 6). Ethanol, being relatively small molecule, finds a way to reach to the active site of CYP3A4 and significantly decreases the docking scores (Table 6). However, our experimental findings that were discussed in earlier sections indicate that presence of COBI in the assays dramatically inhibits CYP3A4 (Figs 3 and 5) giving a little room for ethanol to act on the enzyme. Furthermore, the inability of EVG and COBI to bind simultaneously to CYP3A4 enzyme in the absence or presence of ethanol suggest that docking with two molecules is difficult. However, our experimental data in which COBI masks the EVG metabolism (Fig 3) and CYP3A4 inhibition (Fig 5) suggest that docking does not explain these experimental outcomes. This is not surprising because CYP enzymes especially 3A4 is known to show ligand induced conformational changes and adaption to multiple ligands simultaneously [40, 41]. Overall, these findings suggest that although molecular docking is a good tool to complement the experimental data it cannot fully explain the mechanism of ligand-CYP3A4 interaction that determined through in-vitro study.

In this study, ethanol at physiological concentrations clearly altered the metabolism of EVG and inhibition of CYP3A4 by EVG at physiological concentrations of EVG. As expected, the effect of ethanol in the presence of high concentration of COBI was not apparent. We reasoned that this could be due to ineffectiveness of ethanol when high amount of COBI, a strong inhibitor of CYP3A4, is present, or the influence of ethanol cannot be determined due to strong masking effect of COBI on CYP3A. Nonetheless, this is the first in vitro study for identification of effect of ethanol on EVG metabolism in the absence and presence of COBI. Future analysis of clinical samples from HIV positive alcohol drinkers would give a better understanding of alcohol influence on fixed dose combinations (EVG+COBI) such as Stribild^®^. However, dosing adjustments may be necessary while prescribing EVG alone for instance in the case of Vitekta^®^, once daily EVG tablet that was approved by the FDA for HIV treatment.

As monocytes and macrophages are the key sanctuaries for HIV virus, effectively cleansing of these cells is the primary objective of the antiretroviral drugs. However, it has been reported that efficacy of ART is greatly altered in monocytes and macrophages [42, 43] apparently due to altered cellular pharmacokinetics. In line with this observation, we have previously reported that alcohol exposure to macrophages increases the expression and function of the transporter and CYP enzymes that are involved in the efflux and metabolism of ART, respectively [8, 44]. In view of this established evidence [39], determining the effects alcohol consumption on the efficacy of ART in clinical ex vivo monocytes will be our future goal.

In conclusion, in present study we have shown the evidence for influence of ethanol on the metabolism of integrase inhibitor EVG using a newly developed and validated LC MS/MS method for the first time. In addition, we have shown the influence of ethanol on the inhibitory properties of CYP3A4 by EVG. As integrase inhibitors are increasingly becoming better treatment option for both treatment naïve- and treatment resistant HIV positive people, it is very important to fully understand EVG-alcohol interactions in order to achieve an optimal dosing regimen for the HIV positive alcohol users.

## Supporting Information

S1 File Supporting information that contains original data and/or statistical analysis for Figs 3 to 6. (XLSX)
